# Identification of Novel MicroRNAs and Characterization of MicroRNA Expression Profiles in Zebrafish Ovarian Follicular Cells

**DOI:** 10.3389/fendo.2019.00518

**Published:** 2019-07-31

**Authors:** Yara Zayed, Xin Qi, Chun Peng

**Affiliations:** ^1^Department of Biology, York University, Toronto, ON, Canada; ^2^Centre for Research on Biomolecular Interactions, York University, Toronto, ON, Canada

**Keywords:** microRNAs, RNA-seq, ovarian follicles, follicle development, oocyte maturation, zebrafish

## Abstract

MicroRNAs (miRNAs) are small non-coding RNAs that regulate gene expression primarily at the post-transcriptional levels and thereby play important roles in regulating many physiological and developmental processes. Oocyte maturation in fish is induced by hormones produced from the hypothalamus, pituitary, and ovary. Gonadotropin-releasing hormone (GnRH) stimulates the secretion of luteinizing hormone (LH), which in turn, induces the secretion of maturation-inducing hormone (MIH) from the ovary. It is documented that small early vitellogenic (or stage IIIa) follicles are unable to undergo oocyte maturation whereas oocytes in mid- to late vitellogenic (stage IIIb) follicles can be induced by LH and MIH to become mature. To determine whether miRNAs may be involved in the growth and acquisition of maturational competency of ovarian follicles, we determined the miRNA expression profiles in follicular cells collected from stage IIIa and IIIb follicles using next-generation sequencing. It was found that miRNAs are abundantly expressed in the follicular cells from both stages IIIa and IIIb follicles. Furthermore, bioinformatics analysis revealed the presence of 214 known, 31 conserved novel and 44 novel miRNAs in zebrafish vitellogenic ovarian follicular cells. Most mature miRNAs in follicular cells were found to be in the length of 22 nucleotides. Differential expression analysis revealed that 11 miRNAs were significantly up-regulated, and 13 miRNAs were significantly down-regulated in the stage IIIb follicular cells as compared with stage IIIa follicular cells. The expression of four of the significantly regulated miRNAs, dre-miR-22a-3p, dre-miR-16a, dre-miR-181a-3p, and dre-miR-29a, was validated by real-time PCR. Finally, gene enrichment and pathway analyses of the predicted targets of the significantly regulated miRNAs supported the involvement of several key signaling pathways in regulating ovarian function, including oocyte maturation. Taken together, this study identifies novel zebrafish miRNAs and characterizes miRNA expression profiles in somatic cells within the zebrafish ovarian follicles. The differential expression of miRNAs between stage IIIa and IIIb follicular cells suggests that these miRNAs are important regulators of zebrafish ovarian follicle development and/or oocyte maturation.

## Introduction

Follicle development and oocyte maturation in vertebrates are complex events that require the coordination of hormones originated from the hypothalamus-pituitary-gonadal axis. The hypothalamus produces gonadotropin-releasing hormone (GnRH), which stimulates the secretion of gonadotropins, follicle stimulating hormone (FSH) and luteinizing hormone (LH), from the pituitary gland. In fish, FSH plays a major role in promoting follicle development by inducing estradiol production from ovarian follicular cells while LH acts on follicular cells to induce the production of 17α, 20β-dihydroxy progesterone, known as the maturation-inducing hormone (MIH) (1, 2). MIH then binds to membrane progestin receptors (mPRs), particularly mPRα, expressed on the surface of oocytes (3, 4). This, in turn, activates the maturation promoter factor (MPF), leading to the release of the oocyte from its meiotic arrest and the maturation of the oocyte (5, 6). In addition, many signaling molecules produced within the follicular cells and/or oocytes also act locally to regulate follicle development and oocyte maturation (1, 2).

Prior to engaging in the maturation process, zebrafish follicles develop through three stages. In stage I or the primary growth phase, oocytes begin to grow, and follicles start to form. Stage II is known as the cortical alveolus or previtellogenic stage in which cortical alveoli accumulate within the oocytes. Stage III is characterized by vitellogenesis (7). During this stage, follicles not only increase in size but also develop maturational competency. It has been reported that small early vitellogenic follicles are unable to undergo maturation when treated with human chorionic gonadotropin (hCG, used as an analog of LH), or MIH. In contrast, larger follicles in mid to late vitellogenesis can be induced by these hormones and enter the maturation phase (7, 8). Therefore, the small and mid-late vitellogenic follicles are described as stages III-1 (or IIIa) and III-2 (or IIIb), respectively (8, 9). Signaling molecules produced within the follicles, such as members of the transforming growth factor-β (TGF-β) superfamily, have been shown to regulate maturational competency (10–12).

MicroRNAs (miRNAs) constitute an abundant class of the small, single stranded, non-coding RNA of about 18~26 nucleotides (nt) in length (13–15). In general, miRNAs are first transcribed from intronic or intergenic DNA into primary miRNAs, processed into precursor miRNAs (pre-miRNAs), and then exported into the cytoplasm. The pre-miRNAs have a hairpin structure and further processed into mature miRNA duplexes, which unwind into single-stranded mature miRNAs. In most cases, mature miRNAs interact with the 3′ untranslated region of target mRNAs to reduce their stability and to inhibit translation (15–17). By regulating gene expression, miRNAs are involved in a plethora of developmental and physiological events, including reproduction (18–20). miRNAs have been detected in the ovary of many species, including fish (21–24). It has been reported that miRNAs regulate ovarian functions in mammals, such as granulosa cell proliferation (25) and apoptosis (26, 27), estradiol production (28, 29), and the expression of progesterone (30) and LH/CG receptors (31). A recent study demonstrates that miRNAs are also important regulators of fish reproduction (32). However, the functions of miRNAs during fish follicle development and oocyte maturation are still largely unknown.

We have previously detected miR-17a and miR-430b in zebrafish follicular cells and found that their expression levels are regulated by hCG (33), suggesting that miRNAs may play a role in oocyte maturation. To further investigate whether miRNAs are involved in follicle development and oocyte maturation, especially in the acquisition of maturational competency, we used next-generation RNA sequencing (RNA-seq) to compare the miRNA expression profiles in follicular cells between stage IIIa and IIIb follicles. We identified the significantly regulated miRNAs, predicted novel miRNAs and validated the expression of four of the significantly regulated miRNAs. Finally, through gene enrichment and pathway analyses of predicted target genes of the differentially expressed miRNAs, we identified key pathways that may be important during follicle development and oocyte maturation.

## Materials and Methods

### Animals

Zebrafish were purchased from a local supplier and maintained in 10 L tanks of an AHAB System (Aquatic Habitats, FL) at 28°C, under a 14-h light, 10-h dark cycle. The fish were fed twice a day with commercial tropical fish food. The study protocol was approved by the York University Animal Care Committee. All experiments were performed according to the *Guide to the Care and Use of Experimental Animals* published by the Canadian Council on Animal Care.

### Isolation of Ovarian Follicular Cells

Female zebrafish were anesthetized with 3-aminobenzoic acid ethyl ester (Sigma–Aldrich Canada Inc., Oakville, ON) and decapitated. The ovaries were extracted and maintained in a 100-mm culture dish containing 60% Leibovitz L-15 medium without phenol red (Life Technologies, ThemoFisher Scientific, Burlington, ON). Stage IIIa (0.35–0.51 mm) and IIIb (0.52–0.65 mm) follicles were manually separated and collected according to their size. Follicular cell layers were collected mechanically using fine forceps.

### RNA Extraction and Small RNA Sequencing

Three samples were prepared from each of stage IIIa and IIIb follicular cells for a total of six samples. Each sample contained cells isolated from 120 to 150 follicles pooled from 3 to 4 fish. miRNA-enriched total RNA was extracted using miRNeasy mini kit (Qiagen, Germantown, MD) according to the manufacturer's instructions. The sequencing of small RNAs was performed by the Génome Québec Innovation Center at McGill University using the Illumina HiSeq 2500 Ultra-High-Throughput Sequencing platform.

Raw sequencing reads were processed using the ACGT101-miR program (LC Sciences, Houston, Texas, USA). Adaptor dimers, junk, low complexity, common RNA families and repeats were removed, and only unique sequences of 18–26 nt in length were retained. Unique reads were then mapped to zebrafish precursors obtained from miRBase 22.0. One mismatch within the sequence and length variation at both 3′ and 5′ ends was allowed when aligning reads to zebrafish precursors using BLAST search. Unique reads that were mapped to known precursors were considered known miRNAs. Unannotated reads that mapped to the opposite arm of known pre-miRNAs were considered known miRNAs but were designated either a p3 or p5 depending on whether they were mapped to the 3′ or 5′end, respectively. The remaining sequences were mapped to other selected species precursors in miRBase 22.0 by BLAST search and were designated conserved novel miRNAs. Unmapped sequences were BLASTed against the zebrafish genome version CRCz11, and hairpin RNA structures containing sequences were predicted using RNAfold (34). These predicted miRNAs were considered novel. The following criteria were used to predict the secondary structure of pre-miRNAs: (1) the number of nt in one bulge in stem was ≤ 12; (2) the number of base pairs in the stem region of the predicted hairpin was ≥16; (3) cutoff of free energy (kCal/mol) was ≤ -15; (4) the length of hairpin, up and down stems and terminal loop was ≥50; (5) the length of hairpin loop was ≤ 20; (6) the number of nt in one bulge in the mature region was ≤ 8; (7) the number of biased errors in one bulge in the mature region was ≤ 4; (8) the number of biased bulges in mature region was ≤ 2; (9) the number of errors in mature region was ≤ 7; (10) the number of base pairs in the mature region of the predicted hairpin was ≥12; and (11) the percent of mature region in the stem-loop was ≥80. The results were further refined to only retain miRNAs that met the following criteria: (1) each miRNA should have at least one predicted pre-miRNA and such pre-miRNA should be able to form a hairpin structure, whose genomic coordinates should not overlap with known pre-miRNAs included in this analysis; (2) miRNAs with exact sequence and count matches but had different predicted pre-miRNAs were counted only once; and (3) the retained miRNAs should have at least 100 counts in all sample replicates of either IIIa or IIIb sample sets.

### Differential Expression Analysis, Target Prediction, and Enrichment Analyses

Sequencing counts were first normalized by the library size parameter of the corresponding sample. The differential expression of miRNAs based on the normalized sequencing counts was analyzed using Student's *t*-test (*p* ≤ 0.05) and visualized using Heatmapper (35). Potential targets of the significantly up- or down-regulated miRNAs were predicted by overlapping prediction data of two computational target prediction algorithms: TargetScan (36) and miRanda 3.3a (37). Gene ontology (GO) annotation and Kyoto Encyclopedia of Genes and Genomes (KEGG) signaling enrichment analyses were performed using the ClueGO tool kit (38). The GO terms and KEGG pathways that have a *p*-value ≤0.05 were considered significant.

### Real-Time PCR

miRNA was extracted as described above. The extracted miRNA was polyadenylated and reversely transcribed into cDNA using the NCode miRNA First-Strand Synthesis Kit (Life Technologies) according to the manufacturer's instructions. Real-time PCR (qPCR) was performed using the NCode universal reverse primer along with a forward miRNA-specific primer (Table 1) and EvaGreen qPCR master mix, following the manufacturer's suggested protocol. Relative miRNA levels were determined using the ΔΔ*C*_t_ method after normalization to the endogenous U6 levels.

**Table 1:** List of primers used in real-time PCR.

**miRNA**	**Primer sequence (5^**′**^ to 3^**′**^)**
miR-29a	TAGCACCATTTGAAATCGGT
miR-22a-3p	AAGCTGCCAGCTGAAGAACTGT
miR-16a	TAGCAGCACGTAAATATTGGTG
miR-181-3p	ACCATCGACCGTTGATTGTACC
U6 Forward	CTTGCTTCGGCAGCACATATAC
U6 Reverse	AACGCTTCACGAATTTGCGTG

### Statistical Analysis

Student's *t*-test was used for comparison in miRNA levels between stage IIIa and stage IIIb using GraphPad Prism.

## Results

### Characterization of miRNAs in Zebrafish Ovarian Follicular Cells

**Figure 1:**
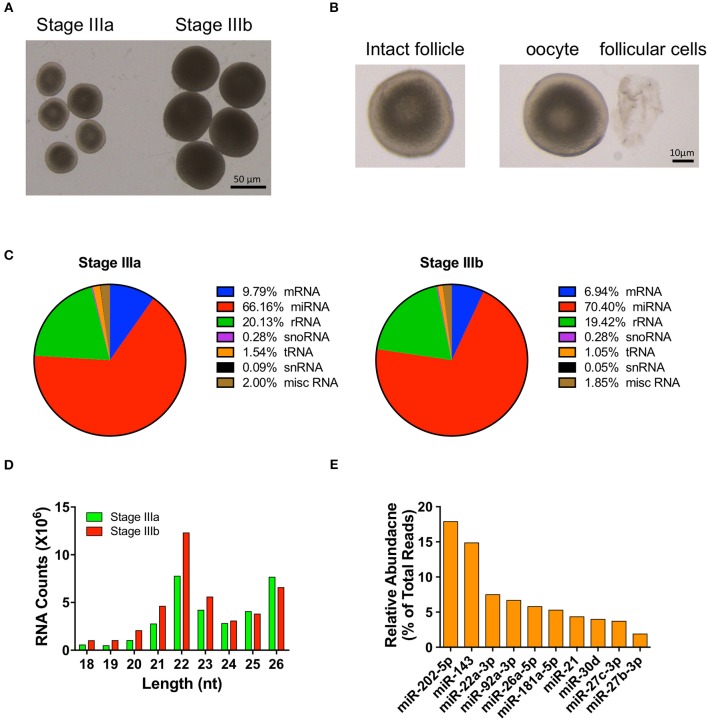
Overview of small RNA-seq data in follicular cells isolated from stage III follicles. **(A)** A picture of stage IIIa and stage IIIb vitellogenic follicles. **(B)** A picture of a stage IIIa follicle before and after follicular cells isolation. **(C)** Distribution of RNA populations in stage IIIa and IIIb follicular cells. **(D)** Read length distribution of small RNA-seq dataset. **(E)** Relative abundance of the 10 most highly expressed miRNAs.

Six cDNA libraries were prepared from miRNAs isolated from three pools of stage IIIa and three pools of stage IIIb follicular cells (Figures 1A,B). Subsequently, Illumina's TruSeq Massively Parallel Sequencing was used to determine the miRNA expression profiles of these samples. After removing low-quality reads, the numbers of mappable reads in stage IIIa and stage IIIb cells were 31601023 and 40205729, respectively. Mapping of the sequencing reads to the zebrafish miRNAs from miRBase 22.0 revealed the presence of 214 known miRNAs. These included 200 annotated miRNAs (Table S1), as well as 9 miRNAs derived from the 3′ arm and 5 miRNAs derived from the 5′ arm of known pre-miRNAs (Table S2). Unmapped reads were then compared to mature miRNA sequences from other species and their genomic locations in the zebrafish genome were determined, resulting in the identification of 31 conserved novel miRNAs (Table S3). The remaining unmapped reads were further mapped to the zebrafish genome and 44 novel miRNAs with predicted precursor miRNAs were identified in zebrafish ovarian follicular cells (Table S4).

Compared to the other RNA populations (i.e., mRNAs, rRNAs, snoRNAs, tRNAs, snRNAs, and other miscellaneous RNAs), the number of miRNAs accounted for 66.16 and 70.40% of the sequencing reads in IIIa and IIIb follicular cells, respectively (Figure 1C). The majority of the mappable miRNA reads detected fell between 21 and 26 nt, with most miRNA reads being 22 nt in length (Figure 1D). Furthermore, the top ten most abundant miRNAs detected were miR-202-5p, miR-143, miR-22a-3p, miR-92a-3p, miR-26a-5p, miR-181a-5p, miR-21, miR-30d, miR-27c-3p, and miR-27b-3p. They constituted 72.64% of the number of mapped miRNAs (Figure 1E) and were expressed in both stage IIIa and IIIb ovarian follicular cells.

### Differential Expression of miRNAs in Stage IIIa and Stage IIIb Follicular Cells

**Figure 2:**
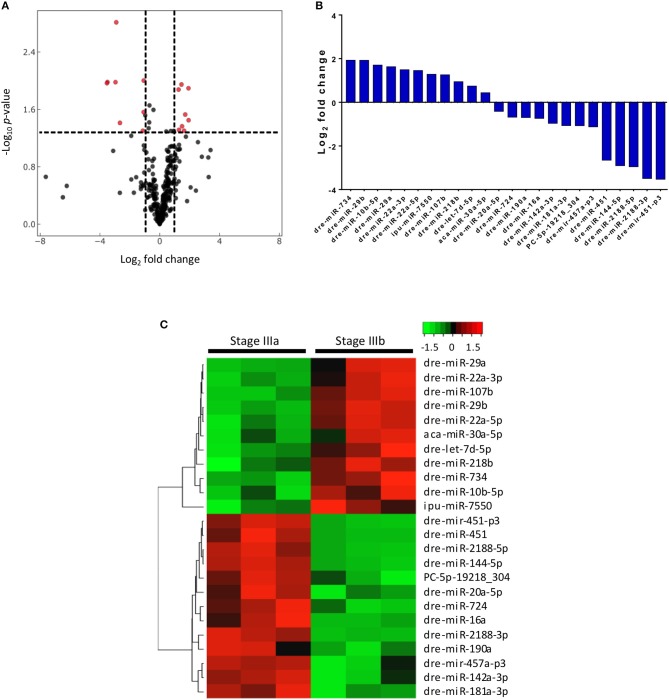
Differential expression of 24 miRNAs in stage IIIa and IIIb follicular cells. **(A)** Volcano plot of miRNAs expressed in stage IIIb vs. stage IIIa follicular cells. Vertical lines indicate fold change <1/2 and >2 and the horizontal line represents the threshold of significant change (*p* ≤ 0.05). **(B)** Relative mean expression of the miRNAs (log_2_ fold change) in stage IIIb follicular cells compared to stage IIIa cells (*P*-value ≤0.05). **(C)** Heatmap of differentially expressed miRNAs between stage IIIa and IIIb follicular cells.

Out of the 289 miRNAs detected in the follicular cells, 24 were differentially expressed between stages IIIa and IIIb, of which 8 were significantly up regulated and 8 were significantly down regulated by more than 2 folds, respectively (Figure 2A). If no limit on the fold change was applied, 9 annotated and 2 conserved novel miRNAs were found to be up-regulated, while 12 annotated and 1 novel miRNAs were down-regulated, in stage IIIb cells when compared with those in stage IIIa (Figures 2B,C).

**Figure 3:**
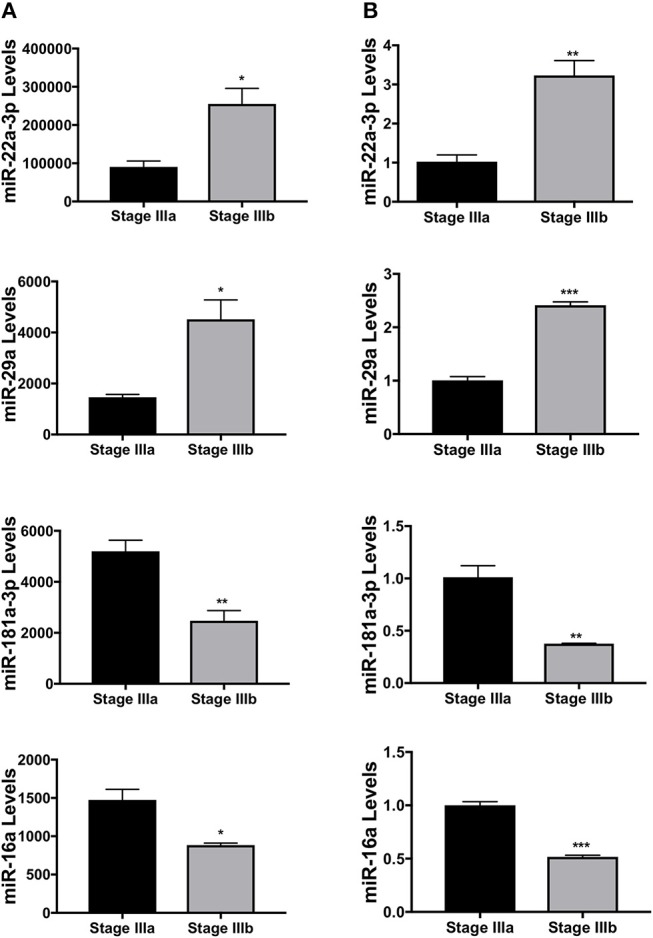
Validation of several miRNAs differentially expressed between stage IIIa and IIIb follicular cells. **(A)** miRNA expression levels of dre-miR-22a-3p, dre-miR-29a, dre-miR-181a-3p, and dre-miR-16a detected by the small RNA-seq. **(B)** Relative expression levels of the same miRNAs measured by real-time PCR. Data represent mean ± SEM (*n* = 3). ^*^P ≤ 0.05; ^**^P < 0.01; ^***^P < 0.001 as analyzed by Student's *t*-test.

Four of the differentially expressed miRNAs identified from the RNA-seq, dre-miR-22a-3p, dre-miR-16a, dre-miR-181a-3p, and dre-miR-29a, were validated using qPCR. New sets of samples were prepared from stage IIIa and IIIb follicles and qPCR was performed. While the trend between the results of sequencing (Figure 3A) and qPCR analyses (Figure 3B) was similar, the qPCR experiments showed a more significant and/or stronger changes in these miRNA levels between the two groups of samples.

### Prediction of Target Genes and Pathway Analyses

**Figure 4:**
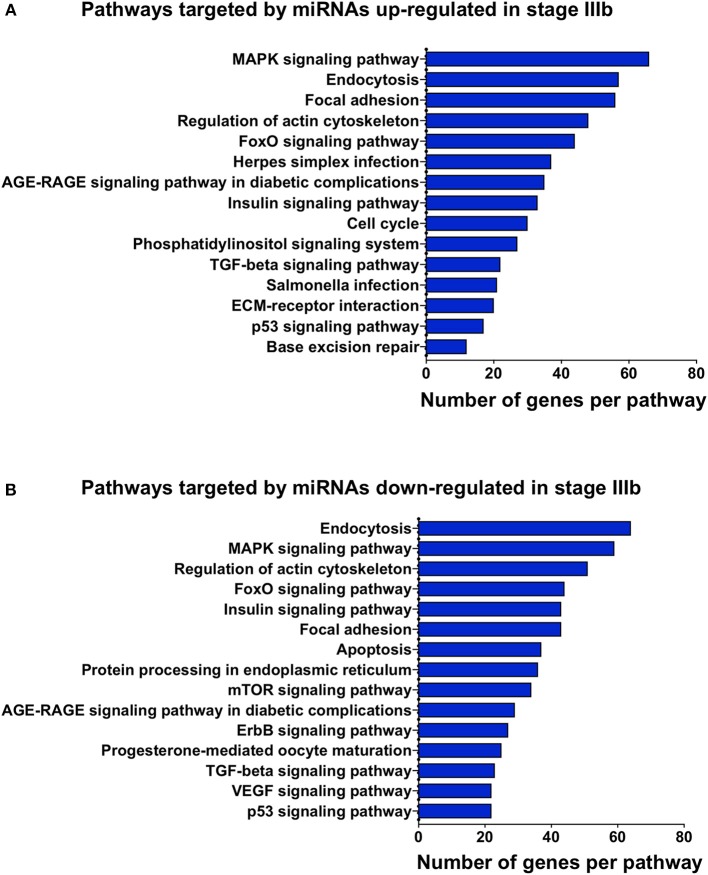
Gene ontology (GO) analysis of the predicted targets of the miRNAs that were differentially expressed between stage IIIa and stage IIIb follicular cells. GO enrichment analysis was performed using ClueGO on the predicted target genes of both up-regulated **(A)** and down-regulated **(B)** miRNAs. X-axis represents the number of target genes while the y-axis shows the terms related to biological processes, molecular function, or cellular components.

**Figure 5:**
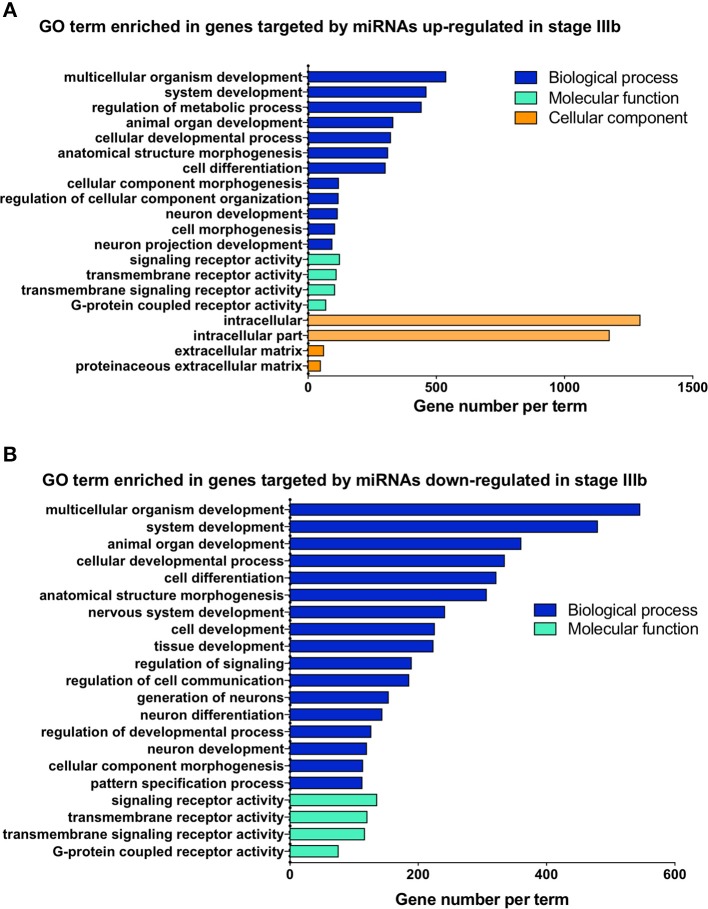
Kyoto Encyclopedia of Genes and Genomes (KEGG) pathway enrichment analysis of the genes predicted to be targeted by up-regulated **(A)** and down-regulated **(B)** miRNAs in stage IIIb follicular cells. KEGG analysis was completed using ClueGO. The x-axis represents the number of target genes and the y-axis denotes the enriched pathway category.

To gain some understanding of the biological functions and signaling pathways regulated by the miRNAs that showed significant changes between IIIa and IIIb follicular cells, we performed GO and KEGG enrichment analyses on the predicted targets of these miRNAs. Most of the biological processes regulated by the differentially expressed miRNAs were related to development (Figure 4). Interestingly, transmembrane receptor activity, signaling receptor activity, and G-protein coupled receptor activity were also amongst the most significantly enriched GO terms (Figure 5). KEGG pathway analysis revealed several key pathways that were enriched in the predicted targets of miRNAs that were differentially expressed between stage IIIa and IIIb cells. Specifically, the MAPK signaling, endocytosis, regulation of actin cytoskeleton, FoxO signaling, insulin signaling, AGE-RAGE signaling, TGF-β signaling, and p53 signaling pathways were significantly enriched in the predicted targets of miRNAs that were both up- and down-regulated in stage IIIb follicular cells. However, cell cycle, herpes simplex infection, salmonella infection, phosphatidylinositol signaling, ECM-receptor interaction, and base excision repair pathways were enriched only in the genes potentially regulated by miRNAs up-regulated in stage IIIb follicular cells (Figure 4A). On the other hand, focal adhesion, apoptosis, mTOR signaling, ErbB signaling, progesterone-mediated oocyte maturation, and VEGF signaling pathways were significantly enriched in the predicted targets of miRNAs down-regulated in stage IIIb. Examination of genes associated with these KEGG pathways (Table S5) revealed that some of the genes were listed under multiple pathways. For example, several genes associated with the MAPK pathway, were also related to FoxO, AGE-RAGE signaling pathway in diabetic complications, and salmonella infection pathway. For pathways that were enriched in the target genes of both up- and down-regulated miRNAs, there were specific genes that were found to be targeted only by the up- or down-regulated miRNAs. Notably, among the TGF-β pathway, ndr1 was targeted by miRNAs down-regulated in stage IIIb follicular cells while tgfb1a, tgfb1b, and tgfb2 were found only in the gene list targeted by miRNAs that were up-regulated in stage IIIb (Table S5). For the MAPK pathway, several fibroblast growth factors (fgf) ligands and receptors, namely fgf13a, fgf3, fgf4, and fgfr2, were only targeted by miRNAs down-regulated in stage IIIb cells (Table S5).

To determine if the miRNAs differentially expressed between stage IIIa and IIIb follicular cells may play a role in oocyte maturation, we further analyzed three key genes known to play critical roles in oocyte maturation, *lhcgr* (encodes LH receptor)*, paqr7b* (encodes mPRα), and *pgrmc1*(encodes progesterone receptor membrane component 1). Among them, pgrmc1 mRNA was the predicted target of miR-451 and miR-144-3p, both were down-regulated by more than 80% in stage IIIb cells. Although the difference in miR-144-3p levels between the two stages were not statistically significant (*p* = 0.095), it was included in this analysis due to the magnitude of its downregulation. On the other hand, lhcgr and paqr7b, were predicted to be targeted by miRNAs that were up- or down-regulated in stage IIIb cells. However, most of the up-regulated miRNAs were present at much lower levels (Table 2).

**Table 2:** Differentially expressed miRNAs that are predicted to target key genes in oocyte maturation.

**miRNA**	**Mean count (IIIA)**	**Mean count (IIIB)**	**Target genes**
dre-miR-142a-3p	484	247	*lhcgr*
dre-miR-144-3p	732	84^*^	*lhcgr, pgrmc1, paqr7b*
dre-miR-16a	1,474	883	*paqr7b*
dre-miR-20a-5p	2,721	2,035	*paqr7b*
dre-miR-451	17,468	2,925	*lhcgr, pgrmc1*
dre-miR-724	2,270	1,723	*lhcgr, paqr7b*
dre-miR-107b	53	127	*paqr7b*
dre-miR-218b	43	82	*lhcgr*
dre-miR-29a	1,459	4,517	*lhcgr*
dre-miR-29b	29	110	*lhcgr*
dre-miR-30a-5p	914	2215	*paqr7b*
dre-miR-734	485	1846	*paqr7b*

* *Not statistically significant between IIIa and IIIb (p = 0.095)*.

## Discussion

The role of miRNAs in fish reproduction is still largely unknown. Using RNA-seq analyses, we characterized miRNAs expressed in follicular cells isolated from ovarian follicles at the stage of vitellogenesis. This led to the prediction of 31 conserved novel and 44 novel miRNAs, thus enhancing our knowledge of miRNAs in zebrafish. In addition, we identified miRNAs that were differentially expressed between stage IIIa follicular cells, which are maturationally incompetent, and stage IIIb follicular cells, which can respond to hormonal signals that induce oocyte maturation. Analyses of predicted target genes of the differentially expressed miRNAs further support the involvement of several key pathways in regulating follicle growth and oocyte maturation and reveal new pathways that can be investigated in future studies.

The most abundant miRNA expressed in the follicular cells of vitellogenic follicles was miR-202-5p, constituting approximately 18% of the total miRNA counts. This miRNA has been shown to be predominantly expressed in the gonads of various vertebrates, including fish (24, 32, 39, 40). The high expression of this miRNA suggests that it is an important regulator of ovarian development and function in vertebrates. In zebrafish, miR-202-5p levels in stage II follicles (pre-vitellogenesis) were strongly up-regulated when compared to stage I (primary growth) follicles (24). Results from this study suggest that this high level of expression is maintained in stage III, during follicle growth and in preparation for oocyte maturation. Thus, it is highly likely that miR-202-5p plays important roles throughout zebrafish follicle development and oocyte maturation. Indeed, a recent study conducted in medaka provides strong evidence to support the critical role of mir-202 in oogenesis/folliculogenesis. Knockout of *mir-202* resulted in the impairment of early follicle development and strongly reduced the number of eggs produced (32). In human, miR-202-5p levels in follicular fluid are positively correlated with the fertilization potential of oocytes (41), suggesting a role for miR-202-5p in the proper development of oocytes. Moreover, miR-202-5p has been shown to regulate PI3K (42) and TGF-β signaling, both of which play important roles in folliculogenesis and oocyte maturation (43, 44).

Two other most abundant miRNAs identified in our study were miR-143 and miR-22a-3p. These miRNAs have also been reported to be highly expressed in mammalian (45) and fish (46–48) ovaries. The role of these miRNAs in fish reproduction has not been reported. However, several studies in mammals suggest that they regulate follicle development. For example, miR-143 has been reported to inhibit the formation of mouse primordial follicles by down-regulating the expression of cell cycle genes and suppressing the proliferation of pre-granulosa cells (49). miR-143 also targets *FSHR*, the gene encoding for the FSH receptor (50), and inhibits granulosa cell proliferation and estradiol production (51). The expression of miR-143 is inhibited by FSH (51) and TGF-β (50). hsa-miR-22-3p, the homolog of dre-miR-22a-3p, was found to be significantly down-regulated in the plasma of patients with premature ovarian insufficiency (52), suggesting a potential role of this miRNA in maintaining proper follicle development. The function and regulation of miR-143 and miR-22a-3p during zebrafish follicle development will be investigated in the future.

In this study, we identified 24 miRNAs that were up- or down-regulated in stage IIIb follicular cells when compared to follicular cells from stage IIIa follicles. Since follicles at stage IIIa are maturationally incompetent, while stage IIIb are capable of responding to hormonal signals and undergo maturation (2), the miRNAs that are differentially expressed between these two stages are likely involved in regulating maturational competency of these follicles. We therefore examined 3 genes that play critical roles in LH- and MIH-induced oocyte maturation to determine if they might be regulated by miRNAs differentially expressed in stage IIIa and IIIb cells. Interestingly, we found that two miRNAs predicted to target *pgrmc1*, whose knockout or inhibition impaired MIH-induced oocyte maturation (53, 54), were strongly down-regulated in stage IIIb cells. It has been reported that oocytes from follicles ranged from 0.55 to 0.65 mm, which are similar to the stage IIIb follicles used in our study, had much higher pgrmc1 protein levels than oocytes at earlier vitellogenic stages (55). Since miRNAs can be secreted and exert paracrine/endocrine regulatory effects on other cells (15), it is possible that the decrease in these two miRNAs contributes to the higher pgrmc1 protein levels observed in oocytes at this stage. Several other differentially expressed miRNAs are predicted to target genes encoding LH receptor and mPRα, which mediate the maturation-inducing effects of LH and MIH, respectively (53, 56, 57). While lhcgr and paqr7b are predicted targets of both up- and down-regulated miRNAs, it was noted that three of the up-regulated miRNAs predicted to target lhcgr or paqr7b had very low levels and therefore may not play a major role in regulating the expression of these genes. Future studies will investigate whether and how these miRNAs target the genes involved in LH and MIH signaling to regulate maturational competency in zebrafish.

Pathway analyses revealed that several key pathways known to be important in regulating oocyte maturation, are among the predicted targets of miRNAs down-regulated in stage IIIb. These include progesterone-mediated oocyte maturation, ErbB, mTOR, and VEGF signaling pathways. In fish, it is well-documented that follicular cells produce 17α, 20β-dihydroxyprogesterone, which acts on membrane progestin receptor to induce oocyte maturation (3). The role of ErbB in oocyte maturation has also been reported in zebrafish (53). A recent study showed that treatment with rapamycin, an mTOR inhibitor, prevented the development of mid and late vitellogenic follicles (58), suggesting that this pathway is important in promoting the growth of follicles from stage IIIa to IIIb. The mTOR pathway has been shown to promote follicle growth (59) and may also be involved in oocyte maturation (60) in mammals. VEGF was reported to enhance the effect of FSH on promoting granulosa cell proliferation (61) and to inhibit ovarian granulosa cell apoptosis (62), suggesting that it plays a role in promoting follicle growth in mammals. Whether and how the VEGF pathway regulates fish follicle development requires further investigation.

One of the most highly enriched pathways targeted by miRNAs differentially expressed between stages IIIa and IIIb follicular cells was the MAPK signaling pathway. This pathway has been well studied in vertebrates and shown to regulate follicle development and oocyte maturation (63–67). In fish, the MAPK pathway has been suggested to mediate the actions of various hormones and growth factors in the ovary, such as regulation of steroid production (68, 69) and activin/inhibin subunit expression (70), as well as oocyte maturation (53, 71). Interestingly, analyses of zebrafish ovarian transcriptomes revealed that the MAPK pathway was also strongly regulated when follicles transitioned from stage I to stage II (72) and most strongly affected by β-diketone antibiotics, which have toxic effects in the reproductive system (73). In this study, we found that this pathway may be targeted by miRNAs either up- or down-regulated in stage IIIb cells. However, some of target genes, such as several fgfs and their receptors, were only found in the genes that may be targeted by miRNAs down-regulated in stage IIIb cells. Although the role of these fgfs in the fish ovary is unknown, several related FGF ligands have been reported to promote oocyte maturation in mammals (74, 75). Together, these findings strongly suggest that the MAPK pathway plays a central role in ovarian follicle development and oocyte maturation and its activity is dynamically regulated by miRNAs.

Many studies have shown that ligands, receptors, and downstream signaling molecules of the TGF-β superfamily are expressed in the zebrafish ovary (76, 77) and that this pathway is involved in early follicle development, follicle growth, and oocyte maturation in zebrafish (10, 70, 78–80). Among the TGF-β family members, activin has potent effects on promoting maturational competency and oocyte maturation (8, 10) while TGF-β1 inhibits oocyte maturation (43, 78, 81). Several bone morphogenetic proteins (BMP), such as BMP-15 (11, 82), BMP2b and BMP4 (83), have been suggested to inhibit precocious oocyte maturation. Consistent with the inhibitory role of TGF-β1 on oocyte maturation, we found that tgfb1a, tgfb1b, and tgfb2 were among the targets of miRNAs up-regulated in stage IIIb follicular cells. However, activin type I and type II receptors (i.e., acvr1ba and acvr2aa) were predicted to be targeted by both up- and down-regulated miRNAs in stage IIIb follicular cells. Smad1 and smad5, which mediate signaling by BMPs, were regulated by the miRNAs down- and up-regulated, respectively, in stage IIIb follicular cells. These findings suggest that activins and BMPs are tightly regulated by miRNAs during vitellogenesis. It is possible that different ligands signaling through these receptors and/or smads have differential effects on regulating follicle growth and/or oocyte maturation.

In conclusion, our study characterizes miRNA expression profiles in follicular cells of zebrafish vitellogenic follicles. We determined the abundance of miRNAs, predicted novel miRNAs, and identified miRNAs differentially expressed between stage IIIa and IIIb follicular cells. Comprehensive gene ontology and pathway enrichment analyses of the predicted targets of the significantly regulated miRNAs revealed several signaling pathways that may be crucial for follicle development and oocyte maturation. Further studies are required to determine how these pathways are regulated by miRNAs and how they are involved in ovarian functions.

## Data Availability

The datasets generated in this study can be found in GEO (https://www.ncbi.nlm.nih.gov/geo/query/acc.cgi?acc=GSE131759).

## Ethics Statement

The animal study was reviewed and approved by York University Animal Care Committee.

## miRNAs Naming Convention

Completely novel miRNAs identified in this study are denoted with a prefix PC-. Conserved novel miRNAs are designated with the name of the species in which the novel zebrafish miRNA has the highest similarity. Newly identified miRNAs that are mapped to a known zebrafish pre-miRNA are tentatively given a name of p3 or p5, depending on whether they are derived from the 3′ or 5′ end of the pre-miRNA stem loop. The naming of these newly identified miRNAs have been submitted to miRbase and their names will be finalized upon the approval of miRBase.

## Author Contributions

YZ and XQ designed and performed the experiments. YZ analyzed the data and drafted the manuscript. CP supervised the study and was involved in experimental design, data analyses, and manuscript writing. All authors approved the submission of the manuscript.

### Conflict of Interest Statement

The authors declare that the research was conducted in the absence of any commercial or financial relationships that could be construed as a potential conflict of interest.
